# Cerebral Aneurysms: A Rare Feature of Behçet's Disease—A Case Report and Review of the Literature

**DOI:** 10.1155/2013/812158

**Published:** 2013-08-01

**Authors:** Samia Younes, Yosra Cherif, Narjes Mokni, Olfa Berriche, Baha Zantour, Amel Boughammoura, Mahbouba Frih-Ayed, Saida Jerbi, Mohamed Habib Sfar

**Affiliations:** ^1^Department of Endocrinology and Internal Medicine, Tahar Sfar University Hospital of Mahdia, Hiboun District, 5100 Mahdia, Tunisia; ^2^Department of Neurology, Fattouma Bourguiba University Hospital of Monastir, 1st June Street, 5000 Monastir, Tunisia; ^3^Department of Medical Imagery, Tahar Sfar University Hospital of Mahdia, Hiboun District, 5100 Mahdia, Tunisia

## Abstract

Behçet's disease (BD) is a multisystem vascular inflammatory disease with several clinical manifestations. Intracranial aneurysms are an extremely rare but nevertheless severe complication of BD. We report a case of a 44-year-old man. The diagnosis of BD was made based on the presence of recurrent oral aphthous ulcers and positive human leukocyte antigen (HLA-) B51 in the absence of evidence of other diseases. MRI showed an ancient ischemic right capsulolenticular lesion, subacute white matter hypersignals of the left capsule lenticular region, and multiple arterial aneurysms. The patient underwent two-month systemic high-dose corticosteroids and immunosuppressive therapy associated with severe neurological deficiency upon admission and severe impairment upon discharge. A thorough review of the literature showed 20 case reports of intracranial aneurysms in BD.

## 1. Introduction

Behçet's disease (BD) is a chronic, relapsing multisystem vascular inflammatory disorder. Vasculitis is its major pathological feature. It mostly affected young adult men [1, 2]. Neurological involvement is relatively uncommon and it may be the first symptom in only 3% of patients [2]. Ischemic events and aneurysms due to Behçet's disease are scarce and may cause life-threatening complications [3–7]. Their pathogenesis remains unknown [2, 8–10]. On account of the risk of serious bleeding complication, it is important to make the diagnosis as soon as possible. We report a rare case of neuro-Behçet in a 44-year-old man with unusual multiple cerebral aneurysms and stroke.

## 2. Case Report

**Figure 1:**
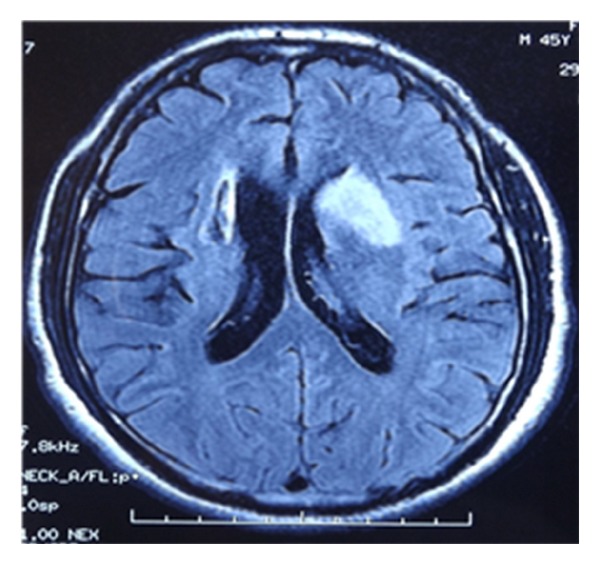
Cerebral MRI axial section diffusion sequence showed bilateral capsule lenticular ischemic lesions (ancient at the right and recent at the left).

**Figure 2:**
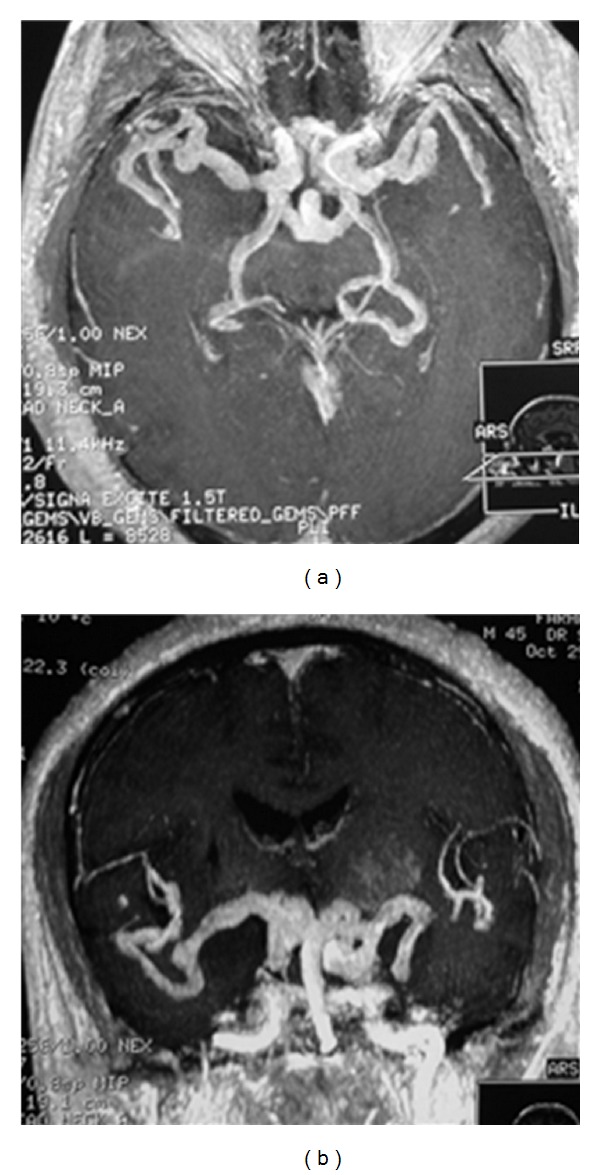
Axial and coronal cerebral MRI T1 gadolinium demonstrated several aneurysms of cerebral arteries.

A 44-year-old right-handed male, with previously healthy status and no family history, was admitted with complaints of acute, generalized headache associated with left arm and leg weakness. He had been suffering from oral aphthous ulcers for 5 years but no genital ulcers. One year ago, he was diagnosed with a cerebral ischemic stroke reveled with left hemiparesis. He developed significant behavioral changes, insomnia, and visual hallucinations that occurred 2 weeks prior to the outbreak of right hemiparesis. The patient was afebrile. He had a blood pressure of 130/80 mmHg, a pulse of 84 beats/min and a respiratory rate of 18 breaths/min. Physical examination revealed also oral aphthous ulceration but no genital scars due to healed ulcers. A thorough neurological examination revealed a normal conscience, no neck stiffness, but dysarthria, quadripyramidal syndrome with tetraparesis, predominant left motor deficit, and bilateral Babinski sign. Examination of the sensibility showed decreased senses of touch, pain, and temperature in the left side of the body. Computed tomography imaging and cerebral MRI disclosed an ancient ischemic right capsulolenticular lesion, with recent ischemic lesions in left capsule-lenticular region (Figure 1). MRI with gadolinium revealed enhancing lesions compatible with intracranial multiple arterial aneurysms (Figure 2). Thoracoabdominal computed tomography angiography had showed no extracranial aneurysms. Laboratory analysis showed a white blood cells count at 9.000 cells/mL with 85% neutrophils, a hemoglobin value at 10.8 g/dL, and a platelet count at 570.000 cells/mL. The erythrocyte sedimentation rate was over 111 mm/hour and the C-reactive protein was at 30 mg/L. Creatinine, electrolyte levels, liver tests, and coagulation studies were within the normal range. All serologic markers of hepatitis B and C, HIV, and syphilis were negative and no microorganisms could be identified from his blood and urine cultures. Immunological analysis including anti-nuclear and anti-phospholipid antibodies, rheumatoid factor, and anti-neutrophilic cytoplasmic antibodies were also within the referential range. Multiple laboratory tests and radiological studies ruled out a hypercoagulability syndrome and a heart disease. A Pathergy test was repeated and found to be clearly negative. The diagnosis of BD was made based on the presence of recurrent oral aphthous ulcers and positive human leukocyte antigen (HLA) B51 in the absence of evidence of other diseases.

Since then, the patient was treated with Methylprednisolone pulses linked with a high-dose regimen of prednisone (1 mg/kg/day) during two months, daily Azathioprine, and physical rehabilitation was started. Coil embolization of aneurysms was not performed because the aneurysms were numerous and there was no bleeding.

**Figure 3:**
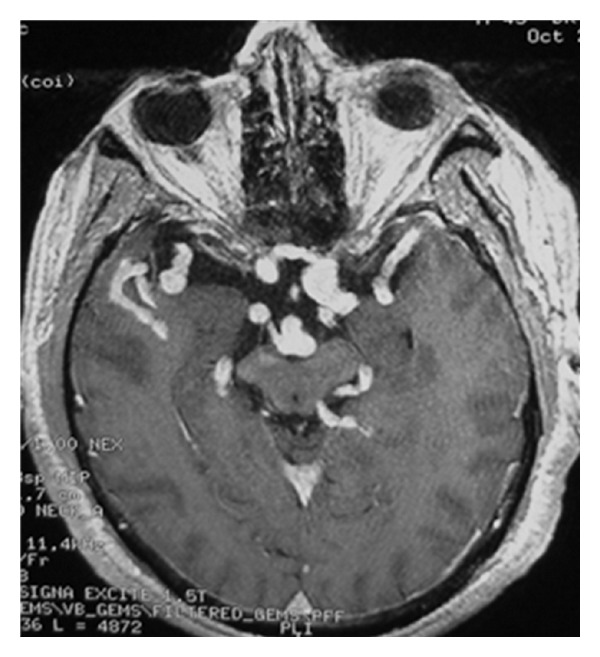
Cerebral MRI showed persistent multiple aneurysms 6 months after treatment.

Ten months after discharge, despite marked regression of mental impairment and no further ischemic events, he developed a pseudobulbar effect including uncontrollable episodes of crying, dysarthria associated with urinary incontinence. The control MRI remained unchanged with persistent diffuse and unruptured aneurysmal dilatation and ancient cerebral stroke (Figure 3).

## 3. Discussion

BD is a multisystemic recurrent inflammatory disorder affecting the eyes, skin and mucosa, joints, vascular system, lungs, gastrointestinal tract, and nervous system [2, 11, 12].

Its clinical pattern and outcomes are various and serious. Venous system's involvement is more common than arterial system's one [2]. The neurological features of BD are mainly related to vasculitis of cerebral vessels. The association of BD with cerebral aneurysms is scarce [2] and its pathogenesis remains unknown.

Peripheral aneurysms are likely more frequent than intracranial even underestimated [2, 11]. Benamour et al. reported only one cerebral aneurysm in 316 cases with BD [1]. Most instances occur, like BD, in men at the age of 41.1 years old (Table 1).

Table 1 summarizes 22 cases of intracranial aneurysms in BD patients previously reported in the literature. The age of these cases was ranged between 12 and 65 years and the male: female ratio was 4.25. The main clinical feature was acute subarachnoid hemorrhage in 12 patients unlike our case of aneurysms first identified as unruptured and associated with an ischemic stroke. The association of cerebral stroke with intracranial aneurysms is rather uncommon detected previously in 4 patients (cases 2, 8, 12, and 14). Cerebral hematoma was detected in 1 patient (case 11). The diagnosis of BD was often established before intracranial aneurysms and the preceding term ranged from 1 month to 25 years (mean: 8 years). Intracranial aneurysms were the first manifestation in 2 cases. Currently, the MRI is considered the most sensitive, noninvasive, and safe screening imaging for the accurate assessment of intracranial aneurysms [2]. Most of them are located in the anterior cerebral circulation, similar to those in the aneurysm cases without BD, and arise from middle cerebral artery (Table 1) and were multiple in 7 cases (Table 1) like our patient. Five of the patients had internal carotid artery aneurysms; 3 had superior cerebellar artery aneurysm (Table 1). Five cases were diagnosed with associated extracranial aneurysms: one retinal aneurysm, another celiac trunk, another superior mesenteric, another with coronary aneurysm, and several pulmonary aneurysms (Table 1). Until it may often be associated with extracranial locations, we might search peripheral arterial involvement which estimated in 7% of patients and it mostly affects the abdominal aorta and the femoral and pulmonary arteries [11]. Furthermore, our review showed peripheral thrombosis in 4 patients with different locations (Table 1). Unlike other cases, our patient had no extracranial aneurysms. Vascular complications such as dissection were mentioned in 2 cases and rupture in 13 cases. One case was associated with arteriovenous malformation; both conditions carry a high risk of delayed bleeding. 

Our review of the literature showed that the cerebral aneurysms were mostly treated in 11 cases with steroid therapy, Colchicine in 3 cases, Cyclophosphamide in 3 cases, Interferon in 1 case, and Azathioprine associated with steroid therapy in our case (Table 1). 

Many attempts were made with clipping in 7 patients and coil embolization in 6 cases and surgical resection was carried out in 2 cases. Given that data is missing for 13 cases, there was satisfactory result in 11 cases after a long-term follow-up; only 2 patients died and relapse of aneurysm of middle cerebral artery occurred in 1 case and was treated successfully with steroid therapy (Table 1). Our patient was treated with a high regimen of corticosteroids. He did not receive antiplatelet drugs because of the risk of major bleeding. He also underwent neither coil embolization nor surgery. 

Vasculitis is more and more raised as the typical pathogenesis in arterial involvement of BD [7, 11, 12], but its part in cerebral aneurysms has not yet been clarified [8–10]. The inflammatory disorder may probably increase the risk of bleeding [13, 14]. Nevertheless, the histopathological examination of 2 cases (1 and 5) with intracranial aneurysms showed no vasculitis [15, 16]. Thus, it suggests the incidental aneurysm in BD, but the aneurysms whether extracranial or intracranial are more common in BD than other vasculitides. This would not be just a coincidence.

The diagnosis of neuro-Behçet can be tricky to establish in the absence of other criteria. Thereafter, the diagnosis of BD in our case was carried on the basis of recurrent oral aphthous ulcers and intracranial aneurysms and was strengthened by the positive human leukocyte antigen (HLA) B51 in the absence of evidence of other diseases. Then, the association of other vasculitides or clinical features at that time may support the diagnosis of BD.

Currently, corticosteroid and immunosuppressive therapy are warranted and beneficial when given in the early stage of the disease to prevent vascular complications and relapses [17]. Because of the multiple aneurysms, their histopathological properties, and the high risk of rupture, surgical resection is performed in carefully selected cases [17]. Many attempts have been made to manage these serious lesions by clipping or coil embolization and may be completely successful. It is difficult to predict its course and response to treatment. Intracranial aneurysms such as in BD carry a poor prognosis despite early and prompt treatment. Intracranial aneurysms rupture and dissection are often the dreadful complication, albeit rare [3, 5, 6, 8, 17–19], associated with a high mortality and poor neurological outcomes [4, 12]. Interestingly, the aneurysm disappears after corticosteroid regimen in several cases [6, 9, 19, 20]. In fact, our patient had no recurrence of cerebral stroke, but aneurysms persist and developed neurological and behavioral impairment upon discharge. 

## 4. Conclusion

Intracranial aneurysm is an extremely rare but nevertheless severe complication of BD. The same lines of treatment used to manage vasculitis in BD and at time endovascular embolization should also be used to treat intracranial aneurysms related to BD. The literature, however, is limited to case reports, and the incidence of these lesions is unknown. It is possible that more of these lesions will be detected with increased use of noninvasive vascular imaging. More work is required to better delineate the natural history of this condition and to come up with treatment guidelines.

## Figures and Tables

**Table 1:** 

Author/Ref.	Sex/Age	Diagnostic delay of BD	Clinical manifestations	Location	Other localizations of aneurysms	Medical treatment	Intervention	Follow-up
Katoh et al. [10]	M/29	/	/	MCA	/	/	Clipping	/
Buge et al. [4]	M/43	8 years	Hemiplegia	ACA, MCA, IC	Retinal an	Corticosteroids	No	/
Kerr et al. [17]	M/12	/	/	MCA, ACom, PCom, AChorA	/	Corticosteroids	Clipping	/
Tsuji et al. [7]	F/62	23 years	Headache, CD, SAH	Bilateral MCA, IC, ACom	No	/	clipping	/
Khodja et al. [8]	M/43	9 years	Lower limb monoparesia	ACom	celiac trunk AN Angiodysplasia AVM	Corticosteroids Colchicine	No	/
Dietl et al. [15]	/	/	/	Bilateral IC	/	Corticosteroids Colchicine	Coil	/
Ildan et al. [21]	M/28	3 years	SAH	ACom	No	Corticosteroids, Cyclophosph.	Clipping	/
El Abbadi et al. [16]	M/44	2 years	CS (MCA)	Bilateral MCA	No	/	Clipping	/
Nakasu et al. [6]	M/57	19 years	SAH, paresia, confusional state	Bilateral MCA	/	/	Clipping	Relapse AN MCA disappeared after steroid therapy
Rosenstingl et al. [19]	M/36	0	SAH	SCER AN	No	Corticosteroids, Cyclophosph. Colchicine	Coil, angioplasty	Disappeared
Koçak et al. [20]	M/37	6 years before	Headache, cerebral hematoma	MCA	No	No	Clipping	Disappeared
Chi and Deruytter [5]	F/43	8 years	Headache, seizures, SAH	SCER AN	No	Received previously corticosteroids Cyclophosph.	Craniectomy	Recovered Died: unknown cause
Itoh et al. [22]	M/65	25 years	Headache, ataxia	Medulla oblongata	No	/	/	/
Kizilkilic et al. [18]	(i) M/38 (ii) M/55	(i) 5 months after (ii) /	(i) SAH, headache (ii) Headache, SAH	(i) SCER AN (ii) PICA	(i) Dissection pseudo an, tapered VA		(i) Coil (ii) Coil	(i) recovered (ii) recovered
Kaku et al. [9]	F/19	1 month	Headache, seizures, SAH, paresis	Bilateral MCA	No	Corticosteroids	Surgery	Recovered
Aktaş et al. [3]	M/38	4 years	Headache, SAH	Trunk of basilar artery	No	No	No	Died
Bahar et al. [23]	M/36	4 years	SAH	IC	SMA AN	Corticosteroids (4 years before)	Coil	Recovered
Agrawal et al. [13]	F/36	6 years	SAH	IC	No	Corticosteroids	Endovascular TRT	Recovered
Senel et al. [14]	M/45	/	SAH	PCA	No	No	Spontaneously thrombosed	Recovered
Okutucu et al. [24]	M/34	0	Headache	MCA	Coronary artery AN	Corticosteroids Cyclophosph. INF	Coil	Recovered
Our case	M/44	0	Hemiplegia	Multiple and bilateral cerebral arteries	No	Corticosteroids Azathioprine	No	Recovered

MCA: middle cerebral artery, ACA: anterior cerebral artery, PCA: posterior cerebral artery, AN: aneurysm, IC: internal carotid, CS: cerebral stroke, CD: consciousness disturbance, SAH: subarachnoid haemorrhage, ACom: anterior communicating artery, AVM: arteriovenous malformation, SCER AN: superior cerebellar artery aneurysm, AChorA: anterior choroidal artery, PCom: posterior communicating artery, PICA: posterior inferior cerebral artery, VA: vertebral artery, SMA: superior mesenteric artery.
